# Antimicrobial Resistance Patterns and Prevalence of *Salmonella* Species in Blood Samples: Insights From a Tertiary Care Center in Nepal

**DOI:** 10.1155/ipid/9167226

**Published:** 2025-11-25

**Authors:** Shiv Kumar Sah, Dhruba Dhungana, Shiva Pandeya, Arjun Budthapa, Deepak Basyal, Balmukunda Regmi, Junu Ricchinbung Rai

**Affiliations:** ^1^Department of Pharmacy, Maharajgunj Medical Campus (MMC), Institute of Medicine (IOM), Tribhuvan University, Kathmandu, Nepal; ^2^Department of Microbiology, Maharajgunj Medical Campus, Institute of Medicine (IOM), Tribhuvan University, Kathmandu, Nepal

**Keywords:** antimicrobial resistance, enteric fever, Nepal, prevalence, *Salmonella*, *Salmonella* Paratyphi

## Abstract

**Background:**

Typhoidal *Salmonella* infections remain a significant public health concern in low- and middle-income countries, with antimicrobial resistance (AMR) posing a major challenge. This study aimed to assess the prevalence and AMR patterns of *Salmonella* species in clinical samples from a tertiary care center in Nepal.

**Methods:**

The study was conducted at the microbiology department in the country's largest public tertiary care hospital, TUTH, Nepal. A retrospective analysis was performed on 16,579 clinical specimens with culture-confirmed *Salmonella* isolates. Antimicrobial susceptibility testing was performed using the Kirby–Bauer disk diffusion method. Resistance and susceptibility trends were analyzed.

**Results:**

Among 16,579 clinical samples analyzed, enteric fever was identified in 114 cases (0.7%). Of these, *Salmonella* Typhi accounted for 77.2%, while *Salmonella* Paratyphi comprised 22.8%. No multidrug resistance was observed, and all isolates were susceptible to third- and fourth-generation cephalosporins (e.g., cefixime, ceftriaxone, and cefepime) and β-lactamase inhibitors (e.g., piperacillin-tazobactam).*Salmonella* Paratyphi showed 100% sensitivity to ampicillin, amoxicillin, and cotrimoxazole, while susceptibility to chloramphenicol was high for both *S.* Typhi (88.9%) and *S.* Paratyphi (92.9%). Gentamicin exhibited comparatively lower sensitivity, with rates of 78.3% for *S.* Typhi and 80% for *S.* Paratyphi. Ciprofloxacin showed the lowest susceptibility, at 47.6% for *S.* Typhi and 60% for *S.* Paratyphi.

**Conclusion:**

Our study identified a lower prevalence of culture-confirmed typhoidal *Salmonella* infections, with *Salmonella* Typhi predominating over *Salmonella* Paratyphi. High resistance to ciprofloxacin and gentamicin was observed, while susceptibility to ampicillin, chloramphenicol, and cotrimoxazole re-emerged, suggesting their potential for reintroduction in treatment. No multidrug resistance or resistance to third- and fourth-generation cephalosporins (e.g., cefixime, ceftriaxone, cefepime) and β-lactamase inhibitors (e.g., piperacillin-tazobactam) was noted, supporting their reliability for empirical therapy. These findings underscore the need for ongoing antimicrobial surveillance to guide effective treatment strategies.

## 1. Introduction

Enteric fever remains a significant global health issue despite advancements in antibiotic therapies and the introduction of newer antibacterial agents. It is a leading cause of morbidity in the developing world, particularly in South Asia, including Nepal [1–3]. Without effective treatment, the case-fatality rate of typhoid fever can range between 10% and 30%, but appropriate therapy reduces this rate to 1%–4% [2]. Despite progress in improving personal hygiene, access to clean water, and sanitation systems, typhoid and paratyphoid fever continue to pose substantial public health challenges [4, 5].

Globally, the incidence rate of typhoid fever in underdeveloped regions of developing countries is estimated to be 55 per 100,000 population [6]. In Nepal, the burden is particularly pronounced in densely populated urban areas and the Terai region, with over 6000 reported cases and more than 100 deaths annually, marking typhoid as a major public health threat [7].

Antimicrobial agents are crucial in preventing complications and fatalities associated with enteric fever. However, the emergence of resistant strains of *Salmonella* Typhi and *Salmonella* Paratyphi has become a major concern [8]. Antimicrobials such as chloramphenicol, ampicillin, and cotrimoxazole were traditionally the drugs of choice for treating enteric fever [9]. However, the extensive and irrational use of these medications has led to the emergence and proliferation of multidrug-resistant (MDR) strains of *Salmonella.* As MDR strains have rendered first-line therapeutic options less effective, fluoroquinolones—particularly ciprofloxacin—became the preferred alternative for treating *Salmonella* infections [10]. Unfortunately, the widespread and indiscriminate use of fluoroquinolones has rapidly led to the emergence of ciprofloxacin-resistant *Salmonella* strains [1]. This resistance necessitated a shift to third-generation cephalosporins, including ceftriaxone, cefixime, and cefotaxime, which are now used more frequently [11]. Over time, resistance has expanded further, moving from first-line drugs to second-line agents, such as fluoroquinolones and third-generation cephalosporins, as well as azithromycin. The trend of resistance shifted from first-line antityphoidal drugs to first- and third-generation cephalosporins and fluoroquinolones (second-line agents for typhoid), and azithromycin [11, 12]. Resistant strains are thus responsible for the failures in treatment [13, 14], narrowing options of drug regimen and are also responsible for increasing the rate of severities and mortalities [15, 16].

In Nepal, the epidemiology of enteric fever has undergone significant changes over the past few decades [17]. However, recent data on the broader impact of this burden on the population remain limited. This lack of comprehensive information highlights the urgent need for routine antimicrobial susceptibility testing of *Salmonella* isolates. Such testing is essential to guide the selection of effective antibiotics and to ensure optimal management of patients with enteric fever. To address this need, our study focuses on determining the prevalence of *Salmonella* species and analyzing their antibiotic resistance patterns among patients at a tertiary care hospital in Nepal. The findings will provide valuable insights into the susceptibility of *Salmonella* species to various antibiotics, helping to develop evidence-based treatment guidelines and strategies tailored to the most recent trends in antimicrobial resistance (AMR).

## 2. Methods

### 2.1. Study Design and Setting

This study was a retrospective, cross-sectional analysis utilizing laboratory reports of culture sensitivity tests for *Salmonella species*. Data spanning a 3 year period, from January 2019 to December 2021, were reviewed to document antimicrobial susceptibility patterns observed during the preceding years.

### 2.2. Study Site and Justification

The study was conducted in the Microbiology Laboratory of Tribhuvan University Teaching Hospital (TUTH), located in Kathmandu, Nepal. TUTH is the largest tertiary care public hospital in the country and serves as a referral center for patients from across Nepal. The hospital's high patient inflow from diverse geographic and socioeconomic backgrounds makes it an ideal site for understanding the epidemiology and (AMR) patterns of enteric fever pathogens on a national scale.

### 2.3. Study Population and Selection Criteria

This study included all eligible clinical specimens received at the Department of Microbiology, (TUTH), during the study period. A total of 16,579 clinical specimens were collected from both in-patient and out-patient Departments for Microbiological culture between January 2019 and December 2021. Among these, 114 cases of enteric fever caused by *Salmonella enterica* were confirmed positive through blood culture.

Patients diagnosed with infections caused by *Salmonella sp.* as well as individuals with available blood culture isolates of *Salmonella*, were considered eligible for inclusion in the study. Data from patients below the age of 1 year were deemed insufficient and were excluded from the analysis.

### 2.4. Variables of Interest

The primary outcome variable of this study was the bacterial isolates. Predictor variables included the antimicrobial susceptibility patterns of these isolates, and demographic information such as the age and sex of the patients. These variables were analyzed to identify correlations and trends, providing insights into the epidemiology and resistance patterns of enteric fever pathogens.

### 2.5. Patients' Characteristics, Specimen Collection, Isolation, and Identification

Required data for this retrospective study were obtained from the records of the Department of Microbiology, TUTH. These records were the result of microbiological procedures as follows: the collection of the blood specimens followed by inoculation in brain heart infusion (BHI) broth maintaining the volume ratio as 1:5, i.e., 5 mL of blood in 50 mL of BHI broth. Aerobic incubation at 37°C was followed by subcultures onto MacConkey agar and blood agar after 24 h and 48 h of incubation. Significant bacterial colonies with suggestive morphologies of *S.* Typhi *and S.* Paratyphi were proceeded for identification by Gram staining and biochemical tests (oxidase test, triple sugar iron test, urea hydrolysis test, citrate utilization test) to confirm the bacterial isolates in accordance with the standard microbiological procedure following the American Society of Microbiology (ASM) [18]. Antimicrobial susceptibility test (AST) of the confirmed isolated was done by the Kirby–Bauer disk diffusion method on Mueller-Hinton agar. Determination of antimicrobials tested in AST and their interpretation was done following the latest Clinical Laboratories and Standard Institute (CLSI) guideline [19]. Any bacterial isolates showing resistance to at least three groups of antimicrobial agents were identified as MDR isolates.

### 2.6. Quality Assurance

The reliability and validity of the study findings were ensured by adhering to standard microbiological procedures recommended by the American Society for Microbiology (ASM) and the Clinical and Laboratory Standards Institute (CLSI) [18]. These guidelines provided a framework for precise and reproducible laboratory practices, including bacterial isolation, identification, and antimicrobial susceptibility testing.

### 2.7. Data Processing and Analysis

Data processing involved systematic verification and cleaning to ensure accuracy and consistency. Data were coded and entered in Microsoft Excel data sheet, and data analysis was done in statistical software, Statistical Package for Social Sciences (SPSS) of version 25.0. Descriptive data were generated. Continuous data were presented in mean ± SD, and frequency (%) tables were generated for categorical variables. Chi-squared test was performed for comparison of categorical variables. All the data were analyzed at 95% CI, and their corresponding 5% margin of error with *p*-value< 0.05 was considered to be statistically significant for all analyses.

The study was conducted in full compliance with the principles outlined in the Declaration of Helsinki. Permission was obtained from the hospital where the study was conducted. Furthermore, the study received ethical approval from the Institute of Medicine-Institutional Review Committee (IOM-IRC) under reference number 294(6-1)E^2^078/079. To safeguard patient confidentiality, all data were anonymized, ensuring that no personally identifiable information was used in the study.

## 3. Results

Table 1 demonstrates the demographic characteristics of the study population. A total of 16,579 patients' blood samples were drawn for microbiological culture. Of these, 114 cases of enteric fever associated with *Salmonella enterica* were confirmed positive by blood culture. Among these, 90.3% of the *Salmonella enterica* isolates were obtained from outpatients, while 9.7% were from inpatients. The mean age of the patients was 21.65 ± 11.10 years (range: 2–90 years), with male patients constituting the majority (56.1%) of those affected by enteric fever. The highest prevalence of enteric fever was observed in the 20–30 age group (41.2%), followed by the 10–20 age group (35.1%). Of the total cases of enteric fever caused by *Salmonella enterica*, 88 (77.2%) were attributed to *Salmonella* Typhi, while 26 (22.8%) were caused by *Salmonella* Paratyphi.

Table 2 illustrates the prevalence of enteric fever within the analyzed population. Out of the 16,579 blood samples tested, the overall prevalence of *Salmonella enteric* fever was estimated at 0.68% (95% CI: 0.58%–0.78%). Among the 114 confirmed *Salmonella* cases, the prevalence of *S.* typhi was 77.19% (95% CI: 69.3%–84.7%), while *S.* Paratyphi accounted for 22.22% (95% CI: 14.4%–29.9%).

Table 3 presents the distribution of *Salmonella* based on patients' characteristics. The prevalence of *S.* typhi (33 cases, 37.5%) and *S.* Paratyphi (14 cases, 53.8%) was higher in the 20–30 age group; however, the detection of the pathogen across different age groups was not statistically significant (*p* > 0.05). Similarly, the distribution of *Salmonella* species showed no significant association with gender (*p* > 0.05).

Table 4 illustrates the antibiogram of *Salmonella* typhi and *Salmonella* Paratyphi isolates. Antimicrobial susceptibility testing, conducted using the Kirby–Bauer disk diffusion method, revealed that all *S.* Typhi isolates (100%) were fully susceptible to cefixime, ceftriaxone, the tazobactam-piperacillin combination, and cefepime. High susceptibility rates exceeding 90% were also observed for ampicillin, amoxicillin, amoxicillin-clavulanic acid, and cotrimoxazole against *S.* typhi.

For *S.* Paratyphi, 100% susceptibility was recorded for ampicillin, amoxicillin, amoxicillin-clavulanic acid, cotrimoxazole, tazobactam-piperacillin, ceftriaxone, cefixime, and cefepime. Chloramphenicol demonstrated high susceptibility, with rates of 88.9% for S. Typhi and 92.9% for *S.* Paratyphi. Azithromycin also exhibited strong efficacy, showing susceptibility rates of 86.7% for *S.* Typhi and 88.9% for *S.* Paratyphi.

In comparison, lower susceptibility was observed for gentamicin, with rates of 78.3% for *S.* Typhi and 80% for *S.* Paratyphi. The lowest susceptibility rates were recorded for ciprofloxacin, at 47.6% for *S.* Typhi and 60% for *S.* Paratyphi.

**Figure 1:**
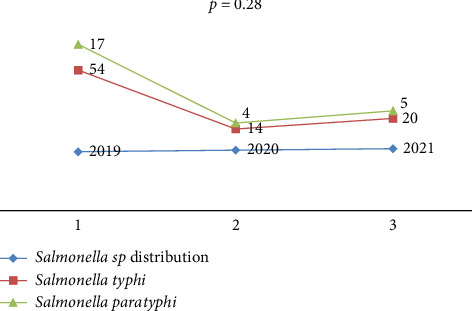
Distributions of *Salmonella* sp. according to year.

Figure 1 demonstrates the distribution of salmonella cases across the study years. The majority of enteric fever cases were recorded in 2019, accounting for 71 cases (62.3%), while fewer cases were observed in 2020 (18 cases, 15.8%) and 2021 (25 cases, 21.9%). However, the variation in case distribution across these years was not statistically significant (*P* > 0.05).

**Figure 2:**
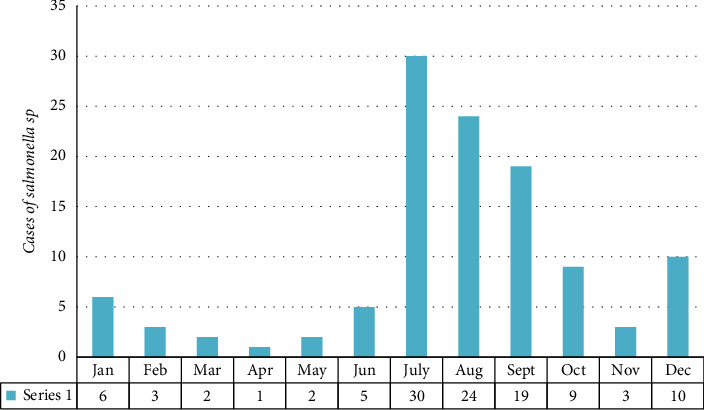
Month-wise distribution of cases of enteric fever.

Figure 2 illustrates the month-wise distribution of *Salmonella* cases. The data reveal the highest prevalence of *Salmonella* infections during the rainy months, with July accounting for the largest number of cases (30, 26.3%), followed by August (24, 21.1%) and September (19, 16.7%).

**Figure 3:**
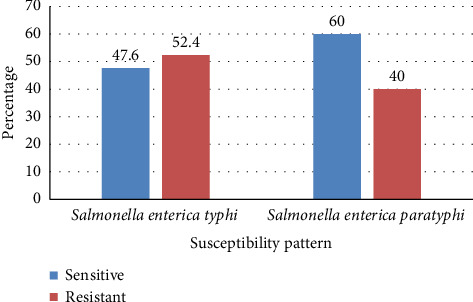
Ciprofloxacin susceptibility pattern of *S. enterica* Typhi *and Salmonella enterica* Paratyphi.

Figure 3 illustrates the susceptibility pattern of ciprofloxacin for *Salmonella* species. Overall, 49.5% of the isolates were resistant to ciprofloxacin. Among the 82 *S. enterica* Typhi isolates, 39 (47.6%) were susceptible, while 43 (52.4%) exhibited resistance. Similarly, among the 25 *S. enterica* Paratyphi isolates, 15 (60%) were susceptible, and 10 (40%) were resistant. Although the difference in susceptibility rates between the two serovars was not statistically significant, *S. enterica* Typhi demonstrated a higher resistance rate (52.4%) to ciprofloxacin compared to *S. enterica* Paratyphi (40%).

## 4. Discussion

Enteric fever remains a major endemic disease in low- and middle-income countries like Nepal. In resource-limited settings, factors such as inadequate access to safe drinking water, poor sanitation, low socioeconomic status, lack of effective surveillance, and weak infection control contribute to the high prevalence of the disease [20]. Nepal faces significant challenges in controlling the disease and addressing rising drug resistance, largely due to the lack of reliable (AMR) data. This study aims to investigate the prevalence of *Salmonella* isolates and their antimicrobial susceptibility patterns in Nepal's largest tertiary care hospital, providing essential insights to address the AMR research gap.

The prevalence of culture-confirmed typhoidal *Salmonella* infection varies significantly across different study settings within the country. For example, a recent study conducted in peri-urban and rural areas of Nepal by Andrews et al. reported a high rate of culture-confirmed typhoidal *Salmonella* infection at 4.1% [21]. Similarly, Raza et al. identified *Salmonella* serotypes in 2.0% of 3980 blood culture samples in their study [22].

In the “Surveillance for Enteric Fever in Asia Project,” an overall incidence of 26% for typhoidal *Salmonella* infections was reported across Bangladesh, Pakistan, and Nepal, with Nepal alone accounting for a 22% incidence of laboratory-confirmed enteric fever cases [23]. In contrast, our study observed a lower prevalence, with culture-confirmed *Salmonella* serotype detected in only 0.68% of the 16,579 clinical samples analyzed.

This variation may be attributed to differences in study recruitment locations. For instance, a study by DO Garrett in Nepal reported laboratory-confirmed enteric fever rates of 17% among outpatients, 2% among inpatients, and 67% from laboratory networks, while 14% were from hospital laboratories, and less than 1% were from surgical wards [23]. In our study, 90.3% of cases were from outpatient departments, which may partially explain the discrepancy.

Additionally, patient-specific characteristics such as age, gender, and seasonal variations could influence these differences. It is also essential to consider the impact of the COVID-19 pandemic during our study period. The adoption of sanitary practices and other preventive measures during the pandemic likely contributed to the reduced prevalence of enteric fever observed in this study.

There has been a persistent shift in the data regarding *Salmonella* serotypes. For instance, Maskey et al. [24] observed a rising trend in *Salmonella* Paratyphi infections, with *S.* Paratyphi A increasing as a proportion of all *Salmonella* isolates from 23.0% during 1993–1998 to 34.0% in 1999–2003. Similarly, Pokhrel et al. [25] reported a higher rate of *S.* Paratyphi (65.4%) compared to *S.* Typhi (34.6%). In contrast, our study found a higher prevalence of *Salmonella enterica* Typhi (77.2%) compared to *Salmonella enterica* Paratyphi (22.8%). This dominance of the Typhi serovar aligns with the findings of Maharjan et al., [9] who reported 75% *S.* Typhi and 27.5% *S.* Paratyphi. Similar trends have also been observed in studies conducted within the country [26] and in neighboring countries [27]. The higher incidence of *S.* Typhi in our study may be attributed to its waterborne mode of transmission, which requires a smaller inoculum compared to the foodborne transmission of *S.* Paratyphi, which typically requires a larger inoculum [28].

The mean age of the population infected with enteric fever was 22 years, aligning with the findings of Khadka et al., who reported a mean age of 22 years [29]. The age group most affected by enteric fever was 20–30 years (41.2%), followed by the 10–20 years group (35.1%). This trend is consistent with the study by Maharjan et al., where the 21–30 age group (47.5%) was the most affected, followed by the 11–20 age group (32.5%) [9]. These observations are further supported by a large-scale study that reported a higher incidence of typhoid in patients aged 16–25 years [23].

Regarding the gender, although there is no association of gender with the incidence of disease, our study showed a higher incidence of cases in male population (56.14%) in comparison to female, which is consistent with reports from Nepal that have consistently demonstrated a higher prevalence of salmonellosis among males compared to that of females [30–33]. This is also supported by the study illustrated in a neighboring country where 44.3% were female and 55.7% were male [27]. In comparison, in earlier studies from Nepal, those with culture-confirmed enteric fever were more often female, with a female to male ratio of 1.4:1 [25].

The inconsistent prevalence of salmonellosis between males and females can be attributed to factors such as increased outdoor exposure among males, leading to greater contact with contaminated sources. Females may have higher prevalence in certain settings due to factors like pregnancy-related vulnerability, immunity differences, and variations in healthcare-seeking behavior. Cultural and regional differences in food consumption, hygiene practices, and healthcare access also contribute to this variability. However, the reason for such discrepancy demands further investigation.

Enteric fever cases can occur year-round, with peaks during the summer and rainy seasons. Consistent with previous reports [9, 34–36], the incidence of typhoid and paratyphoid fever in this study was higher during these periods, with the highest rates observed in July (26.3%) followed by August (21.1%). This indicates that individuals are more vulnerable to the disease during these months, highlighting the need for preventive measures to reduce the risk of infection.

In this study, 62.3% of the cases were reported in 2019, while the prevalence decreased to 15.8% in 2020 and 21.9% in 2021. The decline in enteric fever cases during 2020 and 2021 can be attributed to reduced outdoor activities and limited exposure to sources of infection due to the COVID-19 pandemic. Additionally, fewer people visited hospitals during this period, further contributing to the decreased prevalence.

Our study demonstrated a high susceptibility of *Salmonella* isolates to chloramphenicol (96%), cotrimoxazole (93%), and ampicillin (95.6%), which aligns with the findings of Shrestha et al. [37] who reported susceptibilities of 98.8%, 98.8%, and 97.6% for chloramphenicol, cotrimoxazole, and ampicillin, respectively. Notably, none of the *Salmonella* isolates were MDR, a finding consistent with studies by Chand et al. [26], Maharjan et al. [9], and Khanal et al. [38]. This increased susceptibility might be due to the prolonged discontinuation in the use of traditional antibiotics during the COVID-19 period, the loss of high-molecular-weight resistance plasmids, or due to the emergence of de novo susceptible strains [39].

The increased susceptibility of *Salmonella* to chloramphenicol observed in this study suggests that this antibiotic may still be an effective treatment option for *Salmonella* infections, particularly in regions where resistance to other antibiotics is prevalent. Still, the prudent use of antibiotics and further research into resistance mechanisms should be prioritized to avoid the emergence of resistance and preserve the effectiveness of chloramphenicol for future use.

In this study, 100% susceptibility to cephalosporins (cefixime and ceftriaxone) was observed in all *Salmonella* isolates. This finding is consistent with studies by Gupta et al. [40] and Shrestha et al. [37], both of which reported 100% susceptibility. In contrast, Khadka et al. [29] found a slightly lower susceptibility of 98.9% for both cefixime and ceftriaxone. A study conducted in a neighboring country by Kumar et al. [27] reported a notable prevalence of drug resistance to third-generation ceftriaxone, with 12.1% of *Salmonella* isolates showing resistance.

In our study, both cefixime and ceftriaxone were effective against all ciprofloxacin-resistant *Salmonella* isolates. Additionally, isolates resistant to other antibiotics were also susceptible to these cephalosporins. Third-generation cephalosporins have been widely used to treat MDR and fluoroquinolone-resistant strains [41]. In South Asia, including Nepal, these two antibiotics are currently the primary treatment options for enteric fever [42, 43].

The high susceptibility indicates that third-generation cephalosporins remain an effective therapeutic option for treating *Salmonella* infections, providing clinicians with reliable treatment choices, and should be considered as a first-line treatment for *Salmonella* infections, especially in regions where resistance to older antibiotics is more common.

In this study, ampicillin and amoxicillin demonstrated more than 95% susceptibility against *Salmonella* Typhi and 100% susceptibility against *Salmonella* Paratyphi. Notably, the β-lactamase inhibitor combination, amoxicillin-clavulanic acid, exhibited approximately 90% susceptibility against *S.* Typhi and 100% against *S.* Paratyphi. These findings are consistent with some previous studies that reported a 100% susceptibility of *Salmonella* isolates to amoxicillin [9, 29]. Similarly, Khadka et al. observed a comparable susceptibility rate of 97% [29]. Similarly, β-lactamase inhibitor combination, tazobactum-piperacillin exhibited 100% susceptibility toward salmonella isolates.

The findings of this study have important clinical implications for the treatment of enteric fever caused by *Salmonella* Typhi and *Salmonella* Paratyphi. The observed efficacy of β-lactamase inhibitor combinations, such as amoxicillin-clavulanic acid (approximately 90% for *S.* Typhi and 100% for *S.* Paratyphi) and piperacillin-tazobactam (100% for all isolates), underscores their potential role in overcoming β-lactamase-mediated resistance.

These findings support the continued use of these agents as reliable treatment options, especially in settings where fluoroquinolone resistance is prevalent or macrolide efficacy is declining. However, the slightly lower susceptibility of amoxicillin-clavulanic acid against S. Typhi highlights the need for careful antimicrobial stewardship and susceptibility testing to guide therapy. The exceptional efficacy of piperacillin-tazobactam may position it as a valuable option in severe or MDR cases of enteric fever. Regular surveillance of antimicrobial susceptibility patterns is essential to sustain the effectiveness of these antibiotics and guide clinical decision-making.

A study conducted by Pokhrel et al. [25] in Nepal reported that approximately 78.0% of *Salmonella* Typhi isolates and 88.2% of *Salmonella* Paratyphi isolates were susceptible to ampicillin. Similarly, Chand et al. demonstrated higher susceptibility rates, with 98.2% of *S.* Typhi and 100% of *S.* Paratyphi isolates being susceptible to ampicillin [26]. These findings highlight regional variations in antibiotic susceptibility patterns to these isolates, emphasizing the importance of continuous monitoring of (AMR) patterns to inform treatment guidelines and ensure the effective management of enteric fever.

Incremental drug resistance against fluoroquinolones has been observed over the years, with varying reports from studies conducted in Nepal and neighboring countries [25–27, 44, 45]. In the present study, the emergence of drug resistance to fluoroquinolones was evident. Ciprofloxacin demonstrated reduced susceptibility, with only 47.6% of *Salmonella* Typhi isolates and 60% of *Salmonella* Paratyphi isolates showing sensitivity. Similarly, a study by Laghari et al. in Southern Pakistan reported ciprofloxacin susceptibility rates of 50.1% for S. Typhi and 67.3% for *S.* Paratyphi [46]. These findings align with previous studies in Nepal [38] and neighboring countries [8, 34, 47–50], which have also highlighted the declining sensitivity of ciprofloxacin and other fluoroquinolones.

The growing resistance to ciprofloxacin is concerning and likely multifactorial. Contributing factors may include the overuse of fluoroquinolones due to their cost-effectiveness, accessibility, and oral availability, as well as the widespread availability of antibiotics without a prescription in Nepal [9, 29]. These findings underscore the urgent need for stricter antibiotic stewardship programs, enhanced surveillance of (AMR) patterns, and the implementation of regulatory measures to restrict over-the-counter antibiotic sales. Failure to address these issues could compromise the efficacy of fluoroquinolones as a key therapeutic option for the treatment of enteric fever.

Azithromycin remains a cornerstone in the treatment of enteric fever within the macrolide class of antibiotics. In the present study, the susceptibility of the bacterial isolates to azithromycin was observed to be 87.3%. This rate is notably lower compared to previous studies, including those by Hussain et al. (97%), Khanal et al. (100%), and Laghari et al. (94.6%) [8, 38, 46]. The relatively reduced susceptibility of enteric fever pathogens to azithromycin in this study raises concerns about the potential emergence of (AMR) which could limit the efficacy of azithromycin as a first-line treatment. Continued surveillance and stewardship efforts are essential to preserve the utility of azithromycin in managing enteric fever effectively.

## 5. Limitations

The study was accomplished with some limitations that could be addressed in future studies in similar settings. For instance, the patient's age and gender were only considered as independent variables; however, other sociodemographic and behavioral factors, which might have associated with the resistance pattern, were not examined in the study. Comparisons between hospital-based isolates and community-based isolates were not made. However, the results of this study can help determine the best course of antimicrobial treatment action for a wide range of *Salmonella* spp. Given the cross-sectional design of the study, it precluded any distinction between transitory and persistent isolates.

This study is based on a retrospective analysis of hospital records across different wards; therefore, the available microbiological data did not allow for further subclassification of *Salmonella* Paratyphi into serotypes A, B, or C. In addition, not all isolates were tested against the complete panel of antimicrobials, which limits the generalizability of the susceptibility profile.

Furthermore, the observed variation in prevalence across the study years was likely influenced by the COVID-19 pandemic, which disrupted healthcare access and reporting. Consequently, the figures may not accurately reflect the true year-to-year differences in case prevalence. In addition, the shorter incubation/subculture duration may have influenced the isolation rate.

## 6. Conclusion

Our study demonstrated a lower prevalence of culture-confirmed typhoidal *Salmonella* infections within the study setting, with *Salmonella* Typhi being predominantly more prevalent than *Salmonella* Paratyphi. A seasonal pattern was observed, with the highest infection rates occurring during the rainy months (July, August, and September).

A significant finding was the high rate of resistance among *Salmonella* isolates to ciprofloxacin, followed by gentamicin. However, a notable re-emergence of susceptibility was observed to conventional first-line drugs, including ampicillin, chloramphenicol, and cotrimoxazole. These findings suggest the potential for the reintroduction of these drugs in case management strategies within the study setting.

Importantly, no cases of multidrug resistance were identified in this study. Additionally, no resistance was observed to third-generation cephalosporins (e.g., cefixime and ceftriaxone), fourth-generation cephalosporins (e.g., cefepime), and β-lactamase inhibitor combinations (e.g., piperacillin-tazobactam). These drugs can therefore be considered safe and reliable options for empirical treatment. Furthermore, the restored efficacy of ampicillin, cotrimoxazole, and chloramphenicol highlights their potential as effective alternatives in managing typhoidal *Salmonella* infections.

These findings emphasize the importance of regular antimicrobial surveillance to inform empirical treatment strategies and mitigate the risk of emerging resistance.

## Figures and Tables

**Table 1:** Demographic characteristics of the study population.

Characteristics	Inference
Total clinical samples isolated	16,579
Outpatients	14,970 (90.3%)
Inpatients	1609 (9.7%)
*Salmonella enteric* fever cases	114
Demographic	
Age (years), mean ± SD (minimum-maximum)	21.65 ± 11.10 (2–90)
Gender	
Male	64 (56.10)
Female	50 (43.90)
*Salmonella enterica* from outpatients	90.3%
*Salmonella enterica* from inpatients	9.7%
*Salmonella sp*. (*n* = 114)	
*Salmonella* Typhi	88 (77.2%)
*Salmonella* Paratyphi	26 (22.8%)

**Table 2:** Prevalence of *salmonella* enteric fever.

Total clinical samples (*n* = 16,579)	*Salmonella* spp. types	Prevalence	95% CI (LL-UL)
*Salmonella enteric* fever (*n* = 114)		0.68%	0.58–0.78

Total *Salmonella* spp. (*n* = 114)	*i. S.* Typhi (*n* = 88)	77.20	69.3–84.7
	*ii. S.* Paratyphi (*n* = 26)	22.80	4.4–29.14.4–29.9

**Table 3:** *Salmonella* sp. distribution according to demographics (age and gender) of the patients.

	Causative agent (*n* = 114)		Total	*pvalue*
	*Salmonella* typh*i(n = 88)*	*Salmonella* Paratyphi*(n = 26)*		
Age (continuous)	88 (21.68 ± 12.03)	26 (21.54 ± 7.35)	21.65 ± 11.10	0.95
Age distribution (years)				
1-< 10	11 (12.5%)	1 (3.1%)	12	
10-< 20	31 (35.2%)	9 (34.6%)	40	
20-< 30	33 (37.5)	14 (53.8)	47	
30-< 40	8 (9.1)	2 (7.7%)	10	
≥ 40	5 (5.7)	0 (0.00)	5	
Gender				0.85
Male	49 (55.7)	15 (57.7)	64	
Female	39 (44.3)	11 (42.3)	50	

**Table 4:** Antibiogram of *Salmonella* typhi and *Salmonella* Paratyphi isolates.

Antibiotics		Causative agent	
		*S.* typhi	*S.* Paratyphi
		*n* (%)	*n* (%)
Ampicillin	Sensitive	32 (94.1)	11 (100)
	Resistant	2 (5.9)	0

Amoxicillin	Sensitive	52 (94.5)	15 (100)
	Resistant	3 (5.5)	0

Cotrimoxazole	Sensitive	76 (95)	23 (100)
	Resistant	4 (5)	0

Chloramphenicol	Sensitive	24 (88.9)	13 (92.9)
	Resistant	3 (11.1)	1 (7.1)

Ciprofloxacin	Sensitive	39 (47.6)	15 (60)
	Resistant	43 (52.4)	10 (40)

Cefixime	Sensitive	65 (100)	19 (100)
	Resistant	0	0

Amoxicillin clav.	Sensitive	70 (94.6)	25 (100)
	Resistant	4 (5.4)	0

Tazobactam + Piperacillin	Sensitive	37 (100)	20 (100)
	Resistant	0	0

Ceftriaxone	Sensitive	64 (100)	19 (100)
	Resistant	0	0

Azithromycin	Sensitive	39 (86.7)	16 (88.9)
	Resistant	6 (13.3)	2 (11.1)

Gentamicin	Sensitive	18 (78.3)	4 (80)
	Resistant	5 (21.7)	1 (20)

Cefepime	Sensitive	5 (100)	2 (100)
	Resistant	0	0

## Data Availability

The datasets of the current study will be made available from the corresponding author on reasonable request.

## References

[1] Butt T., Ahmad R. N., Mahmood A., Zaidi S. (2003). Ciprofloxacin Treatment Failure in Typhoid Fever Case, Pakistan. *Emerging Infectious Diseases*.

[2] Crump J. A., Luby S. P., Mintz E. D. (2004). The Global Burden of Typhoid Fever. *Bulletin of the World Health Organization*.

[3] Dewan A. M., Corner R., Hashizume M., Ongee E. T. (2013). Typhoid Fever and its Association With Environmental Factors in the Dhaka Metropolitan Area of Bangladesh: A Spatial and Time-Series Approach. *PLoS Neglected Tropical Diseases*.

[4] Crump J. A., Mintz E. D. (2010). Global Trends in Typhoid and Paratyphoid Fever. *Clinical Infectious Diseases*.

[5] Salerno-Goncalves R., Kayastha D., Fasano A., Levine M. M., Sztein M. B. (2019). Crosstalk Between Leukocytes Triggers Differential Immune Responses Against *Salmonella enterica* Serovars Typhi and Paratyphi. *PLoS Neglected Tropical Diseases*.

[6] Stanaway J. D., Reiner R. C., Blacker B. F. (2019). The Global Burden of Typhoid and Paratyphoid Fevers: A Systematic Analysis for the Global Burden of Disease Study 2017. *The Lancet Infectious Diseases*.

[7] MoHP. Monitoring Evaluation and Operational Research (Meor) (2019). *Nepal Burden of Disease 2017: A Country Report Based on the Global Burden of Disease 2017 Study*.

[8] Hussain A., Satti L., Hanif F. (2019). Typhoidal Salmonella Strains in Pakistan: An Impending Threat of Extensively Drug-Resistant Salmonella typhi. *European Journal of Clinical Microbiology & Infectious Diseases*.

[9] Maharjan A., Dhungel B., Bastola A. (2021). Antimicrobial Susceptibility Pattern of Salmonella spp. Isolated From Enteric Fever Patients in Nepal. *Infectious Disease Reports*.

[10] Rowe B., Ward L. R., Threlfall E. J. (1997). Multidrug-Resistant *Salmonella typhi*: A Worldwide Epidemic. *Clinical Infectious Diseases*.

[11] Butt T., Ahmad R., Salman M., Kazmi S. (2005). Changing Trends in Drug Resistance Among Typhoid Salmonellae in Rawalpindi, Pakistan. *EMHJ-Eastern Mediterranean Health Journal*.

[12] Sharma P., Dahiya S., Manral N. (2018). Changing Trends of Culture-Positive Typhoid Fever and Antimicrobial Susceptibility in a Tertiary Care North Indian Hospital Over the Last Decade. *Indian Journal of Medical Microbiology*.

[13] Ahmad K. A., Khan L. H., Roshan B., Bhutta Z. A. (2011). Factors Associated With Typhoid Relapse in the Era of Multiple Drug Resistant Strains. *The Journal of Infection in Developing Countries*.

[14] Bhutta Z. A., Khan I. A., Shadmani M. (2000). Failure of Short-Course Ceftriaxone Chemotherapy for Multidrug-Resistant Typhoid Fever in Children: A Randomized Controlled Trial in Pakistan. *Antimicrobial Agents and Chemotherapy*.

[15] Kadhiravan T., Wig N., Kapil A., Kabra S., Renuka K., Misra A. (2005). Clinical Outcomes in Typhoid Fever: Adverse Impact of Infection With Nalidixic Acid-Resistant *Salmonella typhi*. *BMC Infectious Diseases*.

[16] Chauhan N., Farooq U. (2021). Multidrug Resistance: A Challenge in Typhoid Treatment. *Asian Journal of Microbiology, Biotechnology and Environmental Sciences*.

[17] Karki S., Shakya P., Cheng A. C., Dumre S. P., Leder K. (2013). Trends of Etiology and Drug Resistance in Enteric Fever in the Last Two Decades in Nepal: A Systematic Review and Meta-Analysis. *Clinical Infectious Diseases*.

[18] Asm Press (2007). *Clinical Microbiology Procedure Handbook*.

[19] Humphries R. M., Ambler J., Mitchell S. L. (2018). CLSI Methods Development and Standardization Working Group Best Practices for Evaluation of Antimicrobial Susceptibility Tests. *Journal of Clinical Microbiology*.

[20] Antillón M., Warren J. L., Crawford F. W. (2017). The Burden of Typhoid Fever in Low-and Middle-Income Countries: A Meta-Regression Approach. *PLoS Neglected Tropical Diseases*.

[21] Andrews J. R., Vaidya K., Bern C. (2018). High Rates of Enteric Fever Diagnosis and Lower Burden of Culture-Confirmed Disease in Peri-Urban and Rural Nepal. *Journal of Infectious Diseases*.

[22] Raza S., Tamrakar R., Bhatt C., Joshi S. (2012). Antimicrobial Susceptibility Patterns of *Salmonella* typhi and Salmonella Paratyphi A in a Tertiary Care Hospital. *Journal of Nepal Health Research Council*.

[23] Garrett D. O., Longley A. T., Aiemjoy K. (2022). Incidence of Typhoid and Paratyphoid Fever in Bangladesh, Nepal, and Pakistan: Results of the Surveillance for Enteric Fever in Asia Project. *Lancet Global Health*.

[24] Maskey A. P., Basnyat B., Thwaites G. E., Campbell J. I., Farrar J. J., Zimmerman M. D. (2008). Emerging Trends in Enteric Fever in Nepal: 9124 Cases Confirmed by Blood Culture 1993–2003. *Transactions of the Royal Society of Tropical Medicine and Hygiene*.

[25] Pokharel P., Rai S., Karki G., Katuwal A., Vitrakoti R., Shrestha S. (2009). Study of Enteric Fever and Antibiogram of Salmonella Isolates at a Teaching Hospital in Kathmandu Valley. *Nepal Med Coll J*.

[26] Chand H. J., Rijal K. R., Neupane B., Sharma V. K., Jha B. (2014). Re-emergence of Susceptibility to Conventional First Line Drugs in Salmonella Isolates From Enteric Fever Patients in Nepal. *The Journal of Infection in Developing Countries*.

[27] Kumar S., Rizvi M., Berry N. (2008). Rising Prevalence of Enteric Fever due to Multidrug-Resistant Salmonella: An Epidemiological Study. *Journal of Medical Microbiology*.

[28] Acharya D., Bhatta D., Malla S., Dumre S., Adhikari N., Kandel B. (2011). *Salmonella* enterica Serovar Paratyphi A: An Emerging Cause of Febrile Illness in Nepal. *Nepal Med Coll J*.

[29] Khadka S., Shrestha B., Pokhrel A., Khadka S., Joshi R. D., Banjara M. R. (2021). Antimicrobial Resistance in *Salmonella* typhi Isolated From a Referral Hospital of Kathmandu, Nepal. *Microbiology Insights*.

[30] Prajapati B., Rai G., Rai S. (2008). Prevalence of *Salmonella typhi* and Paratyphi Infection in Children: A Hospital Based Study. *Nepal Med Coll J*.

[31] Sharma N. P., Peacock S. J., Phumratanaprapin W., Day N., White N., Pukrittayakamee S. (2006). A Hospital-Based Study of Bloodstream Infections in Febrile Patients in Dhulikhel Hospital Kathmandu University Teaching Hospital, Nepal. *Headache*.

[32] Shakya K., Baral M., Shrestha R. (1970). A Study of Atypical Manifestations of Enteric Fever in Childern. *Journal of Nepal Health Research Council*.

[33] Sharma N., Koju R., Karmacharya B. (2004). Typhoid Fever in Dhulikhel Hospital, Nepal. *Kathmandu University Medical Journal*.

[34] Umair M., Siddiqui S. A. (2020). Antibiotic Susceptibility Patterns of *Salmonella* Typhi and *Salmonella* Paratyphi in a Tertiary Care Hospital in Islamabad. *Cureus*.

[35] Sharvani R., Dayanand D., Dayanand D. K., Shenoy P., Sarmah P. (2016). Antibiogram of Salmonella Isolates: Time to Consider Antibiotic Salvage. *Journal of Clinical and Diagnostic Research: Journal of Clinical and Diagnostic Research*.

[36] Malla S., Kansakar P., Serichantalergs S., Rahman M., Basnet S. (2005). Epidemiology of Typhoid and Paratyphoid Fever in Kathmandu: Two Years Study and Trends of Antimicrobial Resistance. *Journal of the Nepal Medical Association*.

[37] Shrestha K. L., Pant N. D., Bhandari R., Khatri S., Shrestha B., Lekhak B. (2016). Re-emergence of the Susceptibility of the *Salmonella* spp. Isolated From Blood Samples to Conventional First Line Antibiotics. *Antimicrobial Resistance and Infection Control*.

[38] Khanal P. R., Satyal D., Bhetwal A. (2017). Renaissance of Conventional First-Line Antibiotics in *Salmonella enterica* Clinical Isolates: Assessment of MICs for Therapeutic Antimicrobials in Enteric Fever Cases From Nepal. *BioMed Research International*.

[39] Khandeparkar P. (2010). Reemergence of Chloramphenicol in Typhoid Fever in the Era of Antibiotic Resistance. *Journal of the Association of Physicians of India*.

[40] Gupta V., Singla N., Bansal N., Kaistha N., Chander J. (2013). Trends in the Antibiotic Resistance Patterns of Enteric Fever Isolates–A Three Year Report From a Tertiary Care Centre. *Malaysian Journal of Medical Sciences: MJMS*.

[41] Effa E. E., Lassi Z. S., Critchley J. A. (2011). Fluoroquinolones for Treating Typhoid and Paratyphoid Fever (Enteric Fever). *Cochrane Database of Systematic Reviews*.

[42] Britto C. D., John J., Verghese V. P., Pollard A. J. (2019). A Systematic Review of Antimicrobial Resistance of Typhoidal Salmonella in India. *Indian Journal of Medical Research*.

[43] Britto C. D., Wong V. K., Dougan G., Pollard A. J. (2018). A Systematic Review of Antimicrobial Resistance in *Salmonella enterica* Serovar Typhi, the Etiological Agent of Typhoid. *PLoS Neglected Tropical Diseases*.

[44] Klemm E. J., Shakoor S., Page A. J. (2018). Emergence of an Extensively Drug-Resistant *Salmonella enterica* Serovar Typhi Clone Harboring a Promiscuous Plasmid Encoding Resistance to Fluoroquinolones and Third-Generation Cephalosporins. *MBio*.

[45] Yanagi D., Vries G. C. d, Rahardjo D. (2009). Emergence of Fluoroquinolone-Resistant Strains of *Salmonella enterica* in Surabaya, Indonesia. *Diagnostic Microbiology and Infectious Disease*.

[46] Laghari G. S., Hussain Z., Hussain S. Z. M., Kumar H., Uddin S. M. M., Haq A. (2019). Antimicrobial Susceptibility Patterns of Salmonella Species in Southern Pakistan. *Cureus*.

[47] Behl P., Gupta V., Sachdev A., Guglani V., Chander J. (2017). Patterns in Antimicrobial Susceptibility of Salmonellae Isolated at a Tertiary Care Hospital in Northern India. *Indian Journal of Medical Research*.

[48] Admassu D., Egata G., Teklemariam Z. (2019). Prevalence and Antimicrobial Susceptibility Pattern of *Salmonella enterica* Serovar Typhi and *Salmonella enterica* Serovar Paratyphi Among Febrile Patients at Karamara Hospital, Jigjiga, Eastern Ethiopia. *SAGE Open Medicine*.

[49] Ali A., Ali H. A., Shah F. H., Zahid A., Aslam H., Javed B. (2017). Pattern of Antimicrobial Drug Resistance of Salmonella typhi and Paratyphi A in a Teaching Hospital in Islamabad. *Journal of Pakistan Medical Association*.

[50] Chiu C.-H., Wu T.-L., Su L.-H. (2002). The Emergence in Taiwan of Fluoroquinolone Resistance in *Salmonella enterica* Serotype Choleraesuis. *New England Journal of Medicine*.

